# Field methods in medical record abstraction: assessing the properties of comparative effectiveness estimates

**DOI:** 10.1186/1472-6963-14-391

**Published:** 2014-09-15

**Authors:** Elizabeth A Cook, Kathleen M Schneider, Jennifer Robinson, June Wilwert, Elizabeth Chrischilles, Jane Pendergast, John Brooks

**Affiliations:** University of Iowa College of Pharmacy, Iowa City, IA USA; Schneider Research Associates, Des Moines, IA 50312 USA; University of Iowa College of Public Health, Iowa City, IA USA; University of South Carolina Arnold School of Public Health, Columbia, SC USA

**Keywords:** Acute myocardial infarction, Medical record abstraction, Medicare, Cardiovascular medications

## Abstract

**Background:**

Comparative effectiveness studies using Medicare claims data are vulnerable to treatment selection biases and supplemental data from a sample of patients has been recommended for examining the magnitude of this bias. Previous research using nationwide Medicare claims data has typically relied on the Medicare Current Beneficiary Survey (MCBS) for supplemental data. Because many important clinical variables for our specific research question are not available in the MCBS, we collected medical record data from a subsample of patients to assess the validity of assumptions and to aid in the interpretation of our estimates. This paper seeks to describe and document the process used to collect and validate this supplemental information.

**Methods:**

Medicare claims data files for all patients with fee-for-service Medicare benefits who had an acute myocardial infarction (AMI) in 2007 or 2008 were obtained. Medical records were obtained and abstracted for a stratified subsample of 1,601 of these patients, using strata defined by claims-based measures of physician prescribing practices and drug treatment combinations. The abstraction tool was developed collaboratively by study clinicians and researchers, leveraging important elements from previously validated tools.

**Results:**

Records for 2,707 AMI patients were requested from the admitting hospitals and 1,751 were received for an overall response rate of 65%; 1,601 cases were abstracted by trained personnel at a contracted firm. Data were collected with overall 96% inter-abstractor agreement across all variables. Some non-response bias was detected at the patient and facility level.

**Conclusion:**

Although Medicare claims data are a potentially powerful resource for conducting comparative effectiveness analyses, observational databases are vulnerable to treatment selection biases. This study demonstrates that it is feasible to abstract medical records for Medicare patients nationwide and collect high quality data, to design the sampling purposively to address specific research questions, and to more thoroughly evaluate the appropriateness of care delivered to AMI patients.

## Background

There is uncertainty about the best combinations of pharmacotherapy for older patients who have experienced an acute myocardial infarction (AMI). To examine the comparative effectiveness of treatments, an examination of the benefits and harms in real world treatment settings is essential [1]. Our larger research objective was to assess the comparative effectiveness of alternative medication treatment combinations after AMI on outcomes such as cardiovascular event-free survival, major side-effect risks, and Medicare costs. We will be estimating the effectiveness of medication combinations by exploiting the real world treatment variation found in Medicare claims databases for patients after an initial AMI. Alternative estimators are available to exploit this variation including risk adjustment (RA) and instrumental variable (IV) approaches; however, the properties of the estimates produced are conditional on assumptions specific to each estimator. Three assumptions must be valid to successfully use instrumental variables: first, the instrumental variable must be relevant and associated with exposure; second, that the variable must affect the outcome only through the likelihood of exposure; and (3) the instrument is unrelated to confounding variables. Under these assumptions, IV estimators yield consistent estimates of treatment effects [2]. Alternatively, observational data can be used to estimate associations of exposures with outcomes, but if exposures are not allocated randomly these associations may be confounded by certain characteristics such as age, severity of illness, or patient frailty. Thus, RA estimators yield unbiased estimates only under this assumption - that unmeasured factors related to treatment choice are unrelated to outcomes – which is difficult to prove [2]. The positive properties of both estimators rely on characteristics of information by definition unmeasured in observational studies. For example, Medicare claims data lack important clinical information about the patient health status, AMI severity, co-existing conditions, and treatment contraindications and complications. As a result, these estimates are vulnerable to treatment selection bias, which arises when physicians tend to treat patients they believe will benefit greatly from pharmacotherapy, and tend not to prescribe medication to those patients where the risks outweigh potential benefits of treatment.

To address this concern, we selected a subpopulation of Medicare AMI patients from our analysis sample and obtained medical records to extract information unmeasured in Medicare claims for these patients. When conducting retrospective studies, in comparison to prospective clinical trials, medical record data are considered the "gold standard" source for relevant and objective clinical information, especially when assessing the effectiveness of medications on outcomes [3].

This paper describes the process and protocol used to collect this supplemental clinical information. We employed a complex sampling strategy, collected extensive data, and relied on hospitals to provide copies of the requested records. Detailed study protocols were developed and adhered to during the course of this project. The aims of this paper are to describe: 1) the permissions required to carry out the study on the Medicare population; 2) the sampling strategy employed to identify records for abstraction; 3) the development and testing of the data collection tool; 4) the data abstraction process and quality assurance, and 5) plans for use of the resulting data.

## Methods

Faculty and staff from the University of Iowa (UI) College of Pharmacy and the College of Public Health, General Dynamics Information Technology (GDIT), and Information Collection Enterprises (ICE) worked collaboratively on this study, entitled "Comparative Effectiveness of Cardiovascular Treatment Combinations Post MI in the Elderly".

### Approvals and protections for obtaining medical records

The study was approved by the Institutional Review Board (01-Biomedical) at University of Iowa (#200908724) in March 2010. Following approval, Medicare claims data were requested from the Centers for Medicare & Medicaid Services (CMS) following the standard process facilitated by the CMS-contracted Research Data Assistance Center (ResDAC). Medicare data used by academic researchers is typically not identified using beneficiary names, Social Security Numbers (SSN) or the Medicare Health Insurance Claim (HIC) numbers. However, this information was necessary for the subsample of patients for whom we desired medical records in order to request the intended records from hospitals.

CMS granted a data use agreement (DUA) for Medicare enrollment and claims data, as well as permission for the collection of medical record data for the study. To ensure that we were able to meet our goal of 1,600 abstracted patient records (determined by the subsampling methodology explained later in this document) we selected a total of 3,072 patients. To remain compliant with The Health Insurance Portability and Accountability Act of 1996 (HIPAA) Privacy Rule (46 CFR Part 160; Part 164 (subparts a,e) [4], CMS approved a process in which ICE acted as an "honest broker", functioning as an entity which received private identifiers from CMS only, unlinked to claims information. To identify the abstraction subsample, we analyzed the de-identified Medicare claims data, selected the cohort, and appended the hospital information necessary to request records - including the address and phone number of the facility as well as admission and discharge dates. A finder file of these AMI patients was sent to the Chronic Condition Data Warehouse (CCW; http://www.ccwdata.org) to have identifiers appended, and the file was then sent to ICE who obtained and abstracted the records without knowledge of the selection algorithm. At the conclusion of the abstraction effort, ICE shared with us the abstracted variables necessary to conduct our work, but none of the private identifiers. Paper copies of the records were shredded at the end of the project; however, the records were scanned and this information will be available until the DUA expires in the event that any data verification is needed. Once the DUA expires, the scanned documents will be destroyed.

### Sampling

We obtained national Medicare enrollment, claims, and Part D prescription drug event data files from the CCW for patients hospitalized with an AMI in 2007 or 2008. We used the standard CCW AMI definition for AMI: an inpatient stay with the primary discharge diagnosis code 410.*x*1 at any time during the year. The acute hospital admission date for each AMI served as the index date for the AMI. The index AMI stay included all Medicare acute and critical access hospital (CAH) claims with overlapping admission and discharge dates following the initial acute hospital AMI admission. We did not consider a patient transferring from one acute or CAH to another to be a separate hospital stay.

We excluded AMIs from our analysis if any of the following conditions were present: 1) the patient did not survive the AMI institutional stay or died within 30 days post discharge; 2) the patient had an AMI in the 12 months prior to the index admission date; 3) the patient was less than 66 years old at the index date to ensure at least one year of Medicare eligibility prior to the index date; 4) the patient was not enrolled in Medicare Part A and Part B fee-for-service during the 12 months prior to the index admission date and the 12 months after the institutional stay discharge date; and 5) the patient was not enrolled in Medicare Part D during the six months prior and the 12 months after the institutional stay discharge date. We further limited the sample by excluding patients who had a Medicare hospice claim during the 12 months prior to the index admission date, and to ensure that we could measure Part D outpatient claims accurately, we required patients to survive 30 days post institutional discharge date without a readmission to a hospital, hospice, or skilled nursing facility [5]. Finally, because we intended to examine area-based treatment rates that were based on driving times, we eliminated patients who resided in Alaska or Hawaii. Our final cohort size was 132,682 post-AMI patients. This cohort served as the source population for abstraction subsampling.

We selected our subsample in a manner to enable us to assess the assumptions underlying RA and IV estimators. We created two key sampling stratification variables:

1. Observed treatment for each of the three study drugs (i.e., statins, beta-blockers, and ACE/ARBs). Each patient was assigned one of eight mutually exclusive treatment groups based on observed Medicare Part D prescription fills during the first 30 days post-discharge: a) No treatment; b) statin alone; c) beta-blocker alone; d) ACE/ARB alone; e) statin + beta-blocker; f) statin + ACE/ARB; g) beta-blocker + ACE/ARB; or h) statin + beta-blocker + ACE/ARB.
2. Area-based treatment ratios (ATRs), a measure of local area prescribing practices. We measured these ATRs at the patient ZIP code-level using the Driving Area of Clinical Care (DACC) method, which required that we calculate driving times and distances between all ZIP codes in the continental U.S [6]. For this analysis, ATRs were determined once a threshold of 200 patients was met in each geographic area [7]. Specifically, we calculated the number of patients with AMI within each ZIP code and if there were fewer than 200, expanded our range to include the next closest ZIP code (using driving time as a measure of proximity) and added those people to our area. This process was repeated until we constructed treatment areas with least 200 people each. The numerator of the ratio was the expected number of people treated in that area, based on the characteristics of the patient and the area (i.e., the covariates), and the denominator was the actual number of people treated. Once the applicable ATRs had been assigned to each patient based on their ZIP code of residence, we created flags to identify "high-use" (those whose ATR values were in the highest quintile) areas corresponding to our eight treatment groups above: a) High "no treatment" area; b) High "statin alone"; c) High "beta-blocker alone"; d) High "ACE/ARB alone"; e) High "statin + beta-blocker"; f) High "statin + ACE/ARB"; g) High "beta-blocker + ACE/ARB"; or h) High "statin + beta-blocker + ACE/ARB". Of the 132,682 patients, 126,236 (95%) fell into one of these high-use ATRs, and became eligible for subsampling. We chose these stratification variables because our resources were limited, and we wanted to obtain patients from areas that offered the most significant contrast in prescribing rates. If we found unmeasured confounders to be similar across these areas, this would provide strong evidence that the distribution of unmeasured confounders was also similar for the areas with rates in-between.

In addition to the two stratification variables, we employed a strategy to ensure geographic balance, to reduce the possibility that our comparisons would reflect idiosyncratic characteristics of local areas. To accomplish this we specified that one-fourth of each stratum for the ATR groups be drawn from each of the four U.S. Census Geographical Regions: Northeast, Midwest, South, and West [8]. A power calculation indicated that a sample size of 200 patients within each of the eight ATR groups (for a total of 1,600) would provide 80% power to detect a difference in mean outcome of 0.36 standard deviation (SD), 85% power to detect a 0.39 SD difference, 90% power to detect a 0.42 SD difference, and 95% power to detect a 0.47 SD difference. Therefore, we designed our project budget to allow us to abstract 1,600 medical records. We created 64 primary sampling units (PSUs) by crossing the eight observed treatments by the eight ATR high-use groups, and drew a stratified random sample, ensuring balance across the four geographic quadrants. A total of 25 abstracted records were desired in each of the 64 PSUs. The sampling design is shown in Table 1.

In anticipation of possible non-response to our initial requests (1,920 cases: 30 per PSU) for medical records, we added a 20% (1,152 cases) oversample to our initial wave of medical record requests [9, 10]. Each sampled AMI stay was given a unique study identification number, to allow for de-identification of the resulting data. Finally, to ensure that this subsample reflected the larger cohort from which it was drawn, we confirmed that the populations were comparable across key variables such as age, sex, race and length of stay (LOS).

**Table 1:** **Sampling design: stratification by observed treatment combinations and area treatment rates**

Treatment combination	High "No treatment" area	High S** area	High *B* area	High **A area	High SB* area	High S*A area	High *BA area	High SBA area	Total
No treatment	25	25	25	25	25	25	25	25	200
(S**)	25	25	25	25	25	25	25	25	200
(*B*)	25	25	25	25	25	25	25	25	200
(**A)	25	25	25	25	25	25	25	25	200
(SB*)	25	25	25	25	25	25	25	25	200
(S*A)	25	25	25	25	25	25	25	25	200
(*BA)	25	25	25	25	25	25	25	25	200
(SBA)	25	25	25	25	25	25	25	25	200
Total	200	200	200	200	200	200	200	200	1,600

S** = statin only; *B* = beta-blocker only; **A = ACE/ARB only; SB* = statin + beta-blocker; S*A = statin + ACE/ARB; *BA = beta-blocker + ACE/ARB; SBA = statin + beta-blocker + ACE/ARB.

### Data collection tool

We created a structured data abstraction tool to obtain information from the medical records of the sampled patients for the index hospital stay, which could have included treatment at two facilities if the patient was transferred during the acute stay. The data collection tool was based loosely on an instrument used for a previous AMI study called the Cooperative Cardiovascular Project (CCP) [11]. We also leveraged key questions from other well-designed medical record abstraction tools such as one used for the Women’s Health Initiative [12] and the Adult Comorbidity Evaluation (ACE-27) [13]. We examined clinical practice guidelines for AMI patients, such as the American College of Cardiology and American Heart Association (ACC/AHA) recommendations [14] and included variables to capture important clinical assessment and treatment information. Variables were modified and customized in consultation with study team cardiologists, internists, and nurses. The domains of information and examples of the types of data elements captured by the medical record abstraction tool are described in Table 2; an exhaustive list of all data elements has not been included due to the excessive number of variables and descriptions. Operational definitions were documented for each variable, including a list of valid sources within a medical record (e.g., admission face sheet, surgical report, medication administration record), inclusion/exclusion criteria, time frame parameters, and medical terms/synonyms. To facilitate the collection of medications on arrival, we imported a list of commonly prescribed medications and their corresponding dosages so that a drop-down list could be offered to facilitate efficiency; the field also allowed for the entry of free text so that medications which did not appear on the drop-down list could be captured.

**Table 2:** **Description of data elements collection in abstraction tool**

**Demographic information**	
•	Diagnosis at admission (STEMI, NSTEMI, unstable angina), arrival setting (home or facility, etc.), primary language
**Presenting symptoms, initial vital signs and tests**	
•	Presentation of chest pain, cognitive status
•	Vitals at admission - pulse, systolic blood pressure, diastolic blood pressure, Respiration, temperature, weight and height
•	Lab tests - LDL, HDL, HA1c, ALT, AST, Serum creatinine, cardiac troponin level, CK-MB, LVEF, ProBNP\BNP
•	ECG findings (left/right ventricular hypertrophy, complete bundle branch block, heart block, AF, other arrhythmia) and Chest x-ray findings
**Prior history – conditions, procedures, hospitalizations, and status immediately prior to AMI admission**	
•	Cardiac conditions and procedures (prior AMI, angina, atrial fibrillation [AF], hypertension, and heart failure [HF]; prior PTCA, coronary artery bypass graft [CABG], stent, or pacemaker,)
•	Non-cardiac condition (liver disease, diabetes, transient ischemic attack [TIA], stroke, chronic kidney disease [CKD], COPD, asthma, dyslipidemia, and tumor)
•	Other history (past/current smoking, drug use, difficulty with activities of daily living and other indicators of mobility limitations and frailty, drug allergies)
**Medication use - prior to admission and in-hospital**	
•	Thrombolytic use, aspirin, clopidogrel/ticlopidine, beta blockers, ACE inhibitors, ARBs, statins, calcium channel blockers, loop diuretic, thiazide diuretics, aldosterone antagonists, digoxin, amiodarone, warfarin, heparin, and other lipid-lowing agents
**In-hospital procedures or complications**	
•	Procedures - coronary angiography and findings, coronary angioplasty, stent placement, echocardiography, and stress test
•	Complications – HF, hypotension, cardiogenic shock, renal failure, major bleed, stroke, pneumonia, sepsis, and pulmonary embolism
**In-hospital labs and test results, and results prior to discharge**	
•	Lab tests (as above), ECG, chest x-ray, stress test, echocardiogram, blood pressure
**Discharge instructions including prescribed medications.**	
•	Cardiovascular-related discharge medications (as above) discharge disposition – to home, skilled nursing, home health, etc.

We anticipated that we might occasionally receive a medical record for a patient who did not meet our study intent (i.e., a patient who was ineligible for secondary prevention with drug therapy), so we included some "stop abstraction" variables in the data collection tool. Incoming records were screened to identify cases disqualified from a full medical record abstraction, which included any of the following: 1) Medical record did not indicate AMI was present on arrival - it occurred at some point later during the hospital stay, 2) AMI was not the primary reason for the admission and may have been secondary to something else such as trauma, 3) AMI was not confirmed by ECG results, 4) AMI occurred, but patient had an invasive non-cardiac procedure within 24 hours of arrival, and 5) AMI occurred, but the medical record indicated that patient was to receive comfort or palliative care only.

The tool was originally created in MS Excel to allow for ease of viewing the dimensions of care, variables, operational definitions, and to facilitate training, review, and revisions of the tool.

After the tool was completed, ICE programmed the data elements into an electronic tool with a user-friendly front end interface using MS Access; this sort of tool had previously been used successfully by this team and others in similar projects [15]. Each case had a study identification number so that it could later be linked with the associated claims data files. No patient names, Social Security numbers, or Medicare HIC numbers were ever included in the database containing the abstracted data.

### Testing and fielding the data collection tool

After pre-testing by a study team physician and the lead abstractor, the instrument was finalized and the lead abstractor trained the remaining four abstractors. Collectively, the abstractors had extensive medical record abstraction experience averaging more than ten years each; all were well-versed in medical terminology, though none were nurses or clinicians. During the training, the study objectives were described, each subsection of the tool was explained, and instructions for each variable were highlighted. Each abstractor received his/her own copy of the abstraction instruction manual and the data dictionary for reference while abstracting the cases. Abstractions were completed at a secure, onsite location exclusively.

Prior to beginning abstraction, all abstractors initially reviewed the same cases, and inter-rater reliability scores were calculated for each data element by comparing each abstractor’s results to the "gold standard" findings from the lead abstractor. All discrepancies were discussed with the abstractors so that eventual data collected would be consistent across the abstractors. Feedback from this training exercise was used to make minor clarifications to the abstraction tool and the manual. When an abstractor demonstrated 95% agreement with the gold standard abstractor for all data elements, s/he began abstracting from the pool of records available. There were 32 data elements in the administrative and demographic portion of the tool, and up to 406 data elements in the rest of the tool, although the number of elements varied by case, depending on the health history and particular clinical management (tests, procedures, complications) of the patient. For example, if a patient had a history of stroke (a "parent" variable), the abstractor checked the "yes" box, and was directed to two "child" variables, regarding the severity and timing of the stroke. If a patient did not have a history of stroke, the abstractor checked the "no" box and was allowed to skip the next two follow-up questions.

### Medical record procurement

The hospital claims data for the abstraction subsample were linked to the Provider of Services (POS) file, a public use file made available by CMS, to obtain hospital contact information for requesting the medical records. ICE mailed a packet to each facility with a patient in the study, that included a cover letter from the Principal Investigator (PI, Brooks), a copy of the CMS DUA to document approval to request medical records, patient-specific identifiers (name, SSN, and HIC), and the admission date of the hospitalization record being requested. The packet also included detailed instructions to photocopy the record and send all components by secure package to ICE. Hospitals were reimbursed a standard amount per record for photocopying and U.S. mail costs.

ICE disseminated record requests for the initial sample (n = 1,920) in April 2012. Since an index hospitalization might be comprised of care at two facilities in the cases of a transfer, there were many instances where more than one record had to be obtained in order to have all the necessary information from the medical record. ICE developed a crosswalk to indicate which cases were comprised of a single medical record and which cases required a set of records. Failure to obtain both parts of a set would render the case un-abstractable.

If the requested medical records from the original sample were not received by ICE, the medical records department was telephoned to re-request the record; in some cases, a copy of the original packet was mailed again. After these efforts to obtain records from the initial sample were exhausted, record requests from the second wave (oversample) were disseminated on an as-needed basis in order to reach a quota of 25 abstracted records per PSU. The record request process was repeated until the required number of records in each PSU was abstracted, or no more records were available to be abstracted.

### Abstraction and quality assurance

Ongoing abstraction began in September 2012 and, initially, aggressive weekly goals of 100 charts per week were established for the abstractors. If, at any time during the data collection process, an abstractor had a question or concern about how to abstract a data element, the on-site lead abstractor was consulted. Questions that required a decision from the clinical team were evaluated (by e-mail or on a weekly call) and responded to within approximately 48 hours. During the abstraction process, our study team clinicians were consulted when a clinical judgment was necessary, rather than requiring that abstractors make clinical judgments.

To maintain quality throughout the entire project, our protocol involved extensive quality control for the abstracted records; re-abstraction of records and inter-rater agreement scores were obtained for 5% of the records abstracted. In addition to the inter-rater agreement process used for all abstractors prior to launching the abstraction data collection effort, ongoing internal quality control (IQC) processes were instituted. Two additional rounds of IQC were performed, both of which occurred after 1/3 of the cases (i.e., approximately 500 records) were abstracted. All rounds of IQC included re-abstraction of a random sample of 25 cases; each of the abstractors completed five cases that were re-abstracted by the lead abstractor. An accuracy-by-variable report was prepared, and abstractors who did not meet the 95% agreement per variable standard were retrained and records they abstracted were re-abstracted by the lead abstractor to ensure accuracy.

To coordinate activities between all study team members and facilitate resolution of any barriers to abstraction, the entire team attended biweekly meetings during which project updates were provided by ICE. These updates included reports detailing the number of records and full sets/cases requested, received, refused, and stopped by PSU. They also highlighted the number of abstracted cases that were complete to date for each PSU.

### Data analysis

To ensure that data were abstracted accurately and uniformly across all members of the abstraction team, results from all three IQC rounds were aggregated by domain (e.g. administrative, lab values, etc. as in Table 2) and agreement with gold standard results was calculated.

Evaluating whether nonresponse bias exists is an important part of assessing the validity of results. Because of the voluntary nature of the response from the facilities, we analyzed the characteristics of patients for whom we received records versus those for whom we did not by linking patient-level beneficiary and Medicare A and B claims from the index AMI stay to our requested and received medical records and examined key dimensions that could bias results (e.g., age, gender, comorbidity history, complications during the stay, length of stay). We also linked data from the CMS POS file to obtain geographic information and additional characteristics of the hospital facilities to allow for comparison of responding versus non-responding facilities.

## Results

**Figure 1:**
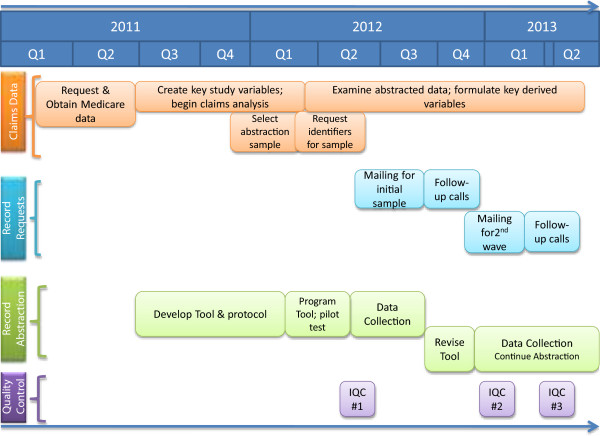
**Timeline of study activities.**

**Figure 2:**
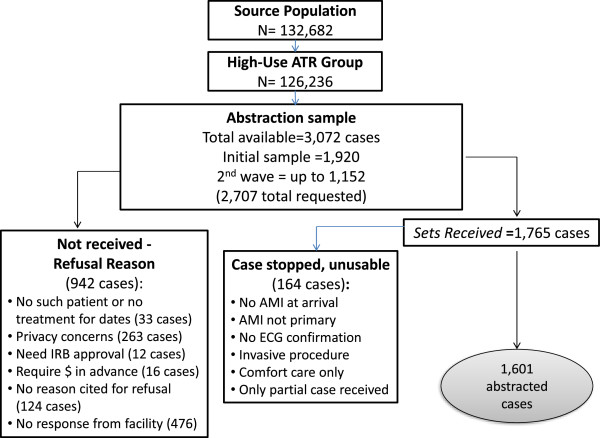
**Sampling.**

The project began in January 2011 when our initial data request for Medicare enrollment and claims data was submitted to CMS for approval, and data collection ended in August 2013 when we met our goal of 1,600 completed medical record abstractions. The actual, rather than intended, timeline for the various study activities is depicted in Figure 1. Many tasks occurred simultaneously. For example, we did not need to have all of the claims data to begin developing the data collection instrument, nor did the instrument need to be finalized before we could begin requesting medical records. Each of these major activities took longer than we anticipated in our initial project plan.A total of 2,707 complete record sets were requested and a total of 1,765 were received for an overall response rate of 65%; of these, a total of 1,601 qualified for abstraction. Of the complete record sets that were received, 164 of them involved at least one of the stop rules. The reasons for stopping the abstraction were, in order of frequency: 1) AMI was not primary reason for admission [120 cases], 2) no confirmation of AMI at arrival [43 cases], 3) no documented ECG within 24 hours of arrival [28], 4) an invasive non-cardiac procedure was performed within 24 hours of arrival [12 cases], and 5) the patient received comfort measures only [11 cases]. More than one stop reason may have been present. For 91 cases, a transfer was involved and the other record was not received, rendering the case unusable for abstraction. Figure 2 documents the medical record sample requested, received, and abstracted.

To reach our goal of 1,600 abstracted records, we eventually relaxed our requirement that each PSU have exactly 25 completed cases. A total of 11 PSUs fell short of our goal of 25 (minimum of 21 cases) and 13 PSUs had more than 25 abstractions (maximum 28 cases); the remaining 40 PSUs had exactly 25 cases abstracted.

The nonresponse analysis revealed that very few differences were found between patients whose records were received versus not received. Examination of claims-based measures at the patient-level indicated that patients for whom we received records tended to be slightly older (79 versus 78 years old at the time of index admission; p < 0.05), were more likely to be discharged home rather than to another facility (57% versus 52%; p < 0.0001), were less likely to require an acute care transfer for the AMI hospitalization (13% versus 21%; p < 0.0001), and had shorter average acute care lengths of stay (LOS) (7.2 days versus 7.9 days; p < 0.05) (Table 3). Other demographics, as well as characteristics of the index AMI, prior comorbid conditions, and the complications of index stay were all comparable.

**Table 3:** **Nonresponse analysis: analysis of beneficiary characteristics**

	Full record received	Full record requested but not received
Number	1,765	942
**Demographics**		
Percent female	59%	56%
Percent dual eligible for Medicaid	32%	31%
Race		
White	83%	84%
Black	6%	7%
Hispanic	7%	6%
Other race	3%	4%
Mean age	79*	78
**Characteristics of AMI**		
Severe/Moderate/Unknown	24%	23%
NSTEMI (not severe)	76%	76%
**Comorbid conditions**		
Unstable angina	30%	33%
Heart Failure	48%	47%
Hypertension	73%	77%
**Complications of index stay**		
Cardiac arrest	3%	2%
Ventricular arrhythmia	8%	8%
Other cardiac arrhythmia	50%	52%
Atrial Fibrillation	21%	19%
Stroke	3%	2%
**Characteristics of hospital stay**		
Multiple acute care facility/transfer	13%***	21%
Discharge disposition		
Discharged home	57%***	52%
Discharged to HH/another facility	43%***	48%
Mean LOS	7.2*	7.9

*p < .05; **p,.001; ***p < .0001.

An examination of the hospital facility-level non-response revealed that we were less likely to receive records from facilities in the Northeast region of the U.S. (17% versus 26%; p < 0.0001) and from facilities in urban [16] areas (77% versus 81%; p < 0.05), but more likely to receive records from the Southern region (38% versus 32%; p < 0.05) of the U.S (see Table 4). We were also less likely to receive records from larger facilities (300+ beds) (42% versus 47%; p < 0.05).

**Figure 3:**
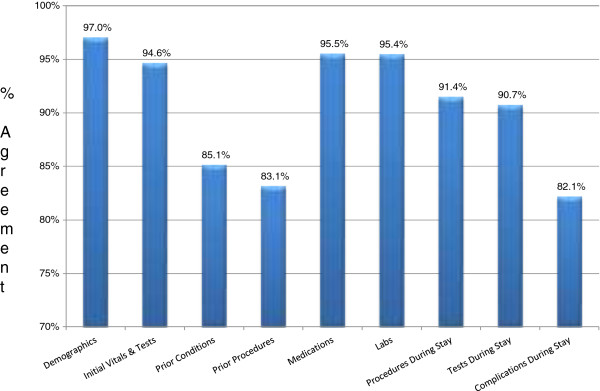
**IQC by domain.**

The abstracted data were of high and uniform quality. There was fairly little variation in the data between abstractors. The overall agreement rate for all three IQC samples was between 95-96%; agreement within each domain ranged from 82.1% (Complications and Conditions During the Stay) to 97% (Administrative and Demographic Information) (see Figure 3). Collecting some of the health history information, for example regarding all prior conditions and procedures, were more challenging for the abstractors, because legibility was often an issue, and some conditions were recorded only once, or found in unexpected places in the record. On average, abstractors spent 45 minutes per abstraction, ranging from a low of 30 minutes to a high of 3 hours.

**Table 4:** **Nonresponse analysis: analysis of facility level characteristics**

Facility characteristics		
	Facility returned one or more records	Facility did not return any records
Number (%)	939 (82%)	200 (18%)
Hospital Type		
For profit	13%	13%
NFP	87%	87%
Number of hospital beds		
Under 100	16%	14%
100-199	24%	22%
200-299	18%	17%
300+	42%*	47%
Region		
Northeast	17%***	26%
Midwest	26%	24%
South	38%*	32%
West	18%	18%
Metropolitan Status		
Urban/Metro	77%*	81%
Nonmetro	23%*	19%

*p < .05; **p < .001.***p<.0001.

## Discussion

Observational comparative effectiveness studies using electronic healthcare data are vulnerable to treatment selection bias. When supplemental data are available for a sample of patients, a variety of methods exist for using these data for sensitivity analyses. For Medicare cohorts, the Medicare Current Beneficiary Survey has been used in this way [17–19]. However, for specific research questions, the MCBS may be too generic to assess critical assumptions and a more focused record abstraction is desirable. We have found that it is possible to efficiently access and abstract medical records and collect high quality data for Medicare patients at a national level to address specific research goals.

Our high agreement level (95-96%) between abstractors for all data elements abstracted supports confidence in our data, as this rate is consistent with standards developed for high reliability in chart review [15]. Moreover, since abstractors who did not meet the 95% agreement per variable standard were retrained and records they abstracted were re-abstracted by the lead abstractor to ensure accuracy, this increases our confidence. During an IQC, we discovered that one abstractor scored very low; therefore, the lead abstractor re-abstracted all of the records she had completed for all elements. We provided additional training for her and closer monitoring until we felt she was proficient in her abstractions. We believe that it is generally preferable to report kappas or intra-class correlation coefficients when quantifying inter-rater agreement for the abstracted data elements; however, we were not able to perform these types of calculations for all items due to concessions we made with regard to the structure of the data collection tool, which did not capture denominators for all items (e.g., grids listing all medications, check only if the item was present). This deliberate decision was made due to a limited budget for abstracting records and a very large amount of desired information from the records.

The overall response rate for obtaining the requested records was reasonable (65%). Similar efforts at medical record procurement have resulted in response rates that range from 44%-94% ( http://www.carecommunications.com; [9, 20]). The beneficiary and facility level non-response analyses suggest that we may have experienced a slight bias towards receiving records for healthier patients, since complicated patients are often treated at or transferred to facilities that have a greater range of services at their disposal, are older and have longer hospitals stays -which result in more lengthy records for photocopying, and are less likely to be discharged home. Hospitals that responded copied an average of 1.85 records per facility; those that did not were asked to copy an average of 1.64; since hospitals were reimbursed a set amount for each record returned, this may have played a role in record procurement. Further analyses are planned to determine whether a weighting strategy is necessary to correct for these differences.

The development of the abstraction tool took much longer than expected, and obtaining the necessary medical records to reach our sample quota was labor-intensive and time-consuming, as others have concluded [15, 21]. However, the protocol was reasonably cost efficient in that it could be accomplished within the budget of a small R01 grant (total grant budget $1.07 M over four years). Some hospitals refused to send records on the grounds that they first required the patient’s permission prior to releasing medical information; in addition, it is possible that a more rapid cycle of follow-up reminders than was used for this study might improve results, as many facilities could not recall receiving a medical record request for our study by the time we made follow-up calls – sometimes several months later. This resulted in having to re-send an entire bulky packet with letters and DUAs; whereas possibly a post card or letter reminder to non-respondents earlier in the process might have been less expensive and potentially resulted in earlier receipt of records. A total of 77 of the cases (about 5%) were abstracted based on PDF files that were created from electronic health records (EHRs). Our abstractors noted that the files that were indexed, such as labs and physician orders, took less time to abstract, because it was easier to find to the correct section and locate the necessary information. Non-indexed PDF files from EHRs did not have this advantage, and took as long as paper records to abstract because one had to navigate each page at a time to find the information. Overall, therefore, the contribution of EHRs to our project progress was minimal, although it was probably easier for hospitals that kept EHRs to respond to our request.

With regard to "stopped" cases, we are aware that for our project, not having a confirmed AMI is a potential confounder when analyzing the effectiveness of pharmacotherapy regimens using Medicare claims data. We plan on assessing the distribution of "stopped" patients between treated and non-treated groups, and between groups based on area treatment rates, to determine whether analytical adjustments will be required.

Our next step is to address the primary objective of evaluating model assumptions and statistically bounding our claims-based estimates of treatment effectiveness. We will estimate the mean differences in each of the construct measures between the patients receiving each of the eight treatment combinations, evaluating the statistical significance of any differences using analysis-of-variance methods (ANOVA).

We are in the process of developing algorithms to assess the severity of AMI, the presence and severity of comorbidities at admission, contraindications to treatment, functional status, the burden of atherosclerosis, and complications during the AMI hospitalization. These algorithms will be tested and validated by comparing to existing approaches in the literature prior to finalization. These measures will serve as a foundation for the next stage of analysis, which will focus on bounding both RA and IV estimates using chart abstraction data to identify unmeasured confounders. For RA estimates, the chart abstraction data will allow us to compare the distributions of unmeasured confounding variables between treated and untreated patients.

These measures will also assist us in the interpretation of our IV estimates. The IV estimator in our study group patients based on ATRs around their residences and exploits the treatment rate differences across these patient groups to yield treatment effect estimates. The underlying assumption in this approach is that unmeasured confounders are evenly distributed across patients grouped by ATRs. To assess the validity of this assumption, we will group the chart abstraction patients based on their local ATRs and compare measures from the chart abstraction between groups. For example, when evaluating the assumptions underlying the incremental benefit of a statin for the patients receiving a statin only, we will compare patients living in areas with high statin-only utilization rates with patients living in areas with high "no treatment" utilization rates. We will use these comparisons to describe potential violations in IV assumptions and the bias directions in our LATE estimates resulting from these violations, and potentially help us "bound" the true LATE effects.

Finally, these data were collected to address specific grant objectives related to unmeasured confounders by our instrument and the treatment received by the patient, but we believe these medical record data will be helpful in addressing other research questions, such as adherence to guideline medications. However, since the patients for whom we obtained data were not randomly selected for abstraction, we plan to use the Medicare data that was used to generate our original abstraction sample to calculate and apply appropriate weights for each abstracted case. Weighting variables will include those used for sampling (ATR values, treatment combinations and geographic region). We will also determine whether any patient demographic, index hospital characteristics, or treatment factors (such as being transferred to another hospital) should be included in the weighting equation. As a result, we should be able to address a wide variety of research questions related to AMI patients and the care they receive during and after their hospital stay.

## Conclusion

Studies using observational data, even when researchers employ instrumental variable methods, are almost always subject to criticism regarding the possibility of treatment selection bias. We believe our methods for collecting, abstracting, and interpreting the important clinical information contained in medical records from Medicare patients provide a valuable approach for researchers to address this issue; not only it is possible to obtain medical records from hospitals on a voluntary basis, but also to collect high quality clinical data to more thoroughly evaluate the effectiveness and appropriateness of care.

## Abbreviations

AMI: Acute myocardial infarction
ACE: Angiotensin-converting-enzyme inhibitor
ARB: Angiotensin II receptor blockers
RA: Risk adjustment
IV: Instrumental variable
UI: University of Iowa
AHRQ: Agency for Healthcare Research and Quality
GDIT: General Dynamics Integrated Technology
ICE: Information Collection Enterprises
CMS: Center for Medicare & Medicaid Services
ResDAC: Research Data Assistance Center
SSN: Social Security Number
HIC: Health Insurance Numbers
HIPAA: Health Insurance Portability and Affordability Act
DUA: Data use agreement
CCW: Chronic Condition Data Warehouse
CAH: Critical access hospital
ATR: Area Treatment Ratio
IRB: Institutional Review Board
SD: Standard Deviation
ANOVA: Analysis of variance
LATE: Local area treatment effect
LOS: Length of stay
NSTEMI: Non ST-elevation MI
STEMI: ST-elevation MI
DACC: Driving Area of Clinical Care
PSU: Primary sampling unit
POS: Provider of Services
IQC: Internal quality control
CCP: Cooperative Cardiovascular Project
LDL: Low-density lipoprotein
HDL: High-density lipoprotein
HA1c: Glycated hemoglobin
ALT: Alanine transaminase
AST: Aspartate aminotransferase
CK-MB: Creatine kinase
LVEF: Left ventricular ejection fraction
ProBNP\BNP: B-natriuretic peptide (BNP)
NT: N-terminal-proBNP
ECG: Electrocardiogram
PTCA: Percutaneous transluminal coronary angioplasty
CABG: Coronary artery bypass graft
ICD: Implantable cardioverter defibrillator
AF: Atrial fibrillation
HF: Heart failure
TIA: Transient ischemic attack
CKD: Chronic kidney disease
COPD: Chronic obstructive pulmonary disease
EHR: Electronic health record.
